# EXAMINING THE IMPACT OF ADVERSE CHILDHOOD EXPERIENCES AND ACTIVE COPING ON COGNITION IN OLDER AFRICAN AMERICANS

**DOI:** 10.1093/geroni/igae098.0786

**Published:** 2024-12-31

**Authors:** Karen Graham, Audrey Steinman, Olimpia Paun, Lisa Barnes

**Affiliations:** Rush University Alzheimer’s Disease Research Center, Chicago, Illinois, United States; University of Illinois Chicago Hospital and Health Sciences System, Chicago, Illinois, United States; Rush University, Chicago, Illinois, United States; Rush University Medical Center, Chicago, Illinois, United States

## Abstract

**Objective:**

To examine the association between Adverse Childhood Experiences (ACEs) and cognition in older African Americans and to determine whether active coping moderates the effects of ACEs on cognition later in life. Design: Secondary analysis of data from the Minority Aging Research Study (MARS), a longitudinal community-based study that examines cognitive health and aging in older African Americans.

**Methods:**

Data from 756 MARS participants were analysed. Participants completed cognitive global functioning tests as well as questionnaires to evaluate exposure to ACEs (The First 18 Years Survey) and active coping (John Henryism Active Coping Scale). We used a correlational analysis (t-tests, ANOVA, linear regression) to examine the associations between ACEs and cognition. Additionally, active coping (an inner ability to address difficult experiences) was examined to determine if it moderated the impact of ACEs on cognition.

**Results:**

ACES were not associated with global cognitive functioning. However, a negative relationship was found between active coping and global cognitive functioning, such that more active coping was associated with lower cognition. We did not find active coping to be a significant moderator of ACEs. Conclusions: High active coping scores were found to be correlated to impaired cognitive functioning. More research is needed to address this phenomenon as well as the impact of ACEs on older Black people.

